# Determination of Sexual Dimorphism of the Human Sacrum Based on Receiver Operating Characteristic Curve Analysis of Morphometric Parameters

**DOI:** 10.7759/cureus.38629

**Published:** 2023-05-06

**Authors:** Sandeep Saluja, Sarika R Tigga, Sushant S Das, Avinash Thakur

**Affiliations:** 1 Anatomy, Lady Hardinge Medical College and Associated Hospitals, New Delhi, IND; 2 Anatomy, Amrita School of Medicine, Faridabad, IND; 3 Anatomy, Employees’ State Insurance Corporation (ESIC) Medical College and Hospital, Alwar, IND; 4 Anatomy, All India Institute of Medical Sciences, Bilaspur, Bilaspur, IND; 5 Anatomy, Employees’ State Insurance Corporation (ESIC) Medical College, Faridabad, IND

**Keywords:** sexual dimorphism, sex estimation, sacral index, sacra, roc analysis, morphometric parameters

## Abstract

Background

Sex estimation of unidentified incomplete skeletons poses a challenge to paleoanthropologists and forensic experts. The sacrum is a part of the axial skeleton and contributes to the formation of the pelvic girdle. It is a significant bone for the identification of the sex in the human skeletal system due to associated functional differences of the pelvic bones in males and females. However, there is a lack of cognizance of different morphometric parameters of the sacrum which may be crucial for determining sex, particularly when a part of the bone is available. This study aimed to recognize the best morphometric parameters for the identification of the sex of the sacrum even when fragmented bones were available and compare the various parameters for sexual dimorphism in different populations.

Methodology

The study was conducted on 110 dry adult human sacra in the anatomy department. Out of these, 42 sacra were female and 68 were male. Morphometric measurements were performed with the help of a digital vernier caliper. Statistical analysis was performed using SPSS version 17.0 (SPSS Inc., Chicago, IL, USA). Morphometric measurements of male and female sacra were compared using Student’s t-test. The receiver operating characteristic (ROC) curve analysis was performed to establish the most appropriate cut-off values for each parameter.

Results

The mean sacral length measured from the promontory to the apex of the sacrum was higher in males compared to females (p < 0.001), whereas the sacral index was higher in female sacra in comparison to male sacra (p < 0.001). Furthermore, the mean height of the first posterior sacral foramina (PSF) was higher in male sacra bilaterally (p < 0.05). On ROC analysis, the area under the curve was 0.994 for the sacral index and 0.862 for the sacral length.

Conclusions

In this study, the sacral index was noted to be the most important morphometric parameter for the identification of the sex of the sacra. Additionally, the height of the S2 body, the height of the first anterior sacral foramina, and the height of the first PSF can be contemplated with an accuracy of 60-70% if only a part of the sacrum is available for determining the sex. Hence, this study emphasizes the significance of morphometric parameters of the sacrum in the determination of sex, especially in forensic cases when the skull and pelvis are fragmented or unavailable.

## Introduction

Determining the sex of unknown skeletons poses a challenge to paleoanthropologists, paleodemographers, and medicolegal experts [1]. Various methodologies for sex determination on different bones of the human skeleton have been investigated [2]. Among several bones of the human body, pelvic bones are the most significant for the identification of sex as the functions of these bones differ in both sexes [3]. These differences may be attributed to the varying stature and hormonal difference in both sexes. Furthermore, in females, the pelvic bones are structurally adapted to facilitate childbirth [4]. Morphometric determination of sex from pelvic bones becomes particularly vital when only partial or fragmented remains are available where the morphology is equivocal [5]. Among pelvic bones, the sacrum is an important bone for the estimation of sex in the human skeletal system. It is a confluence of five progressively smaller sacral vertebrae and their costal elements, sustaining the stability of the vertebral column [6]. The sacrum has always fascinated forensic experts and anthropologists for the identification of sex because of allied functional sex variances [5].

Davivongs described that male bones are generally larger and heavier than female bones [7]. However, sex estimation by visual impressions of the bones can be less valid because of the many drawbacks associated with the subjective assessment of the investigators. Recently, the factual morphometric measurement in different bones has become the basis for sexual dimorphism [1].

Different investigators had studied various morphometric parameters of the human sacra but a single and effective measurement to identify sex has not been established so far. Consequently, it is recommended to utilize the maximum possible parameters to establish the sex of the sacrum. Many studies have investigated sexual dimorphism wherein sacra of known sex were studied and the authenticity of the parameters was validated. Morphometric measurement of dry human sacra is preferable compared to radiographs as it eradicates the restrictions of conventional radiographs, such as inconsistency in the film-focus distance, movement of the spine, and parallax effect [5].

The sacrum is often contemplated as a crucial bone when deciding the sex of unknown skeletal material. Still, there is a lack of knowledge regarding various morphometric parameters of the sacrum which may be vital for determining sex, especially when fragmented bones are available [5]. This study attempted to identify the best morphometric parameters for the identification of the sex of sacra even when it is partly broken and compare the various morphometric parameters for sexual dimorphism in different populations.

## Materials and methods

A cross-sectional, observation study on dry adult human sacra was conducted in the anatomy department. Of the 110 dry adult human sacra, 68 were male and 42 were female. Sacra showing damage, mutilation, or anomalies that might affect the measurement and sacra of indeterminate sex were excluded from the study. Linear measurements were performed with the aid of a digital vernier caliper with a precision of up to 0.01 mm. Two measurements were performed for a parameter and an average was determined. In this study, the below-mentioned parameters were taken into consideration.

**Figure 1:**
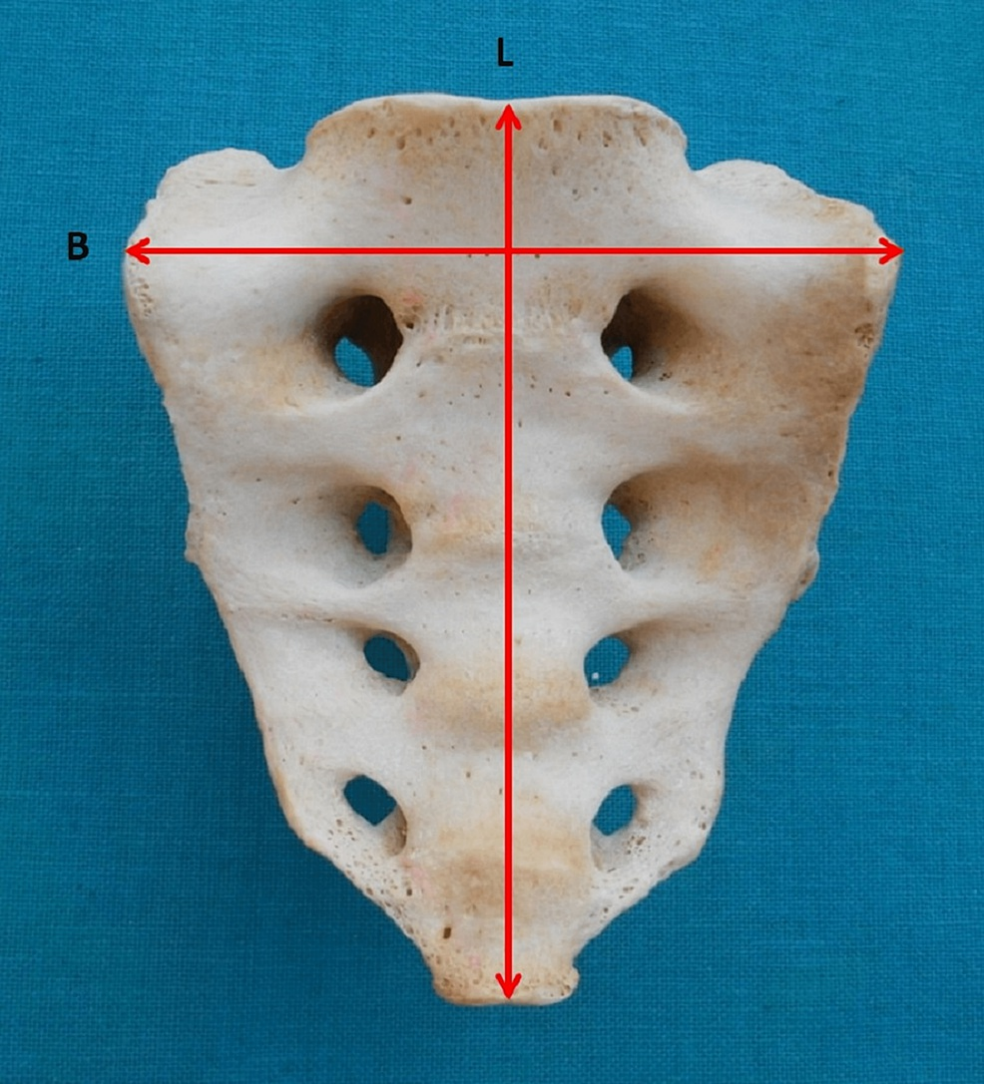
Anterior surface of the sacrum. L = length from the promontory to the apex; B = maximum breadth across the base

Maximum breadth across the base referred to the maximum breadth between two points at the superior part of the auricular surface (Figure 1).

Length from the promontory to the apex was the maximum length measured from the mid-point of the promontory to the mid-point of the inferior border of the last sacral vertebra (Figure 1).

The sacral index was calculated using the following formula: Maximum breadth across the base/Length from promontory to apex × 100.

**Figure 2:**
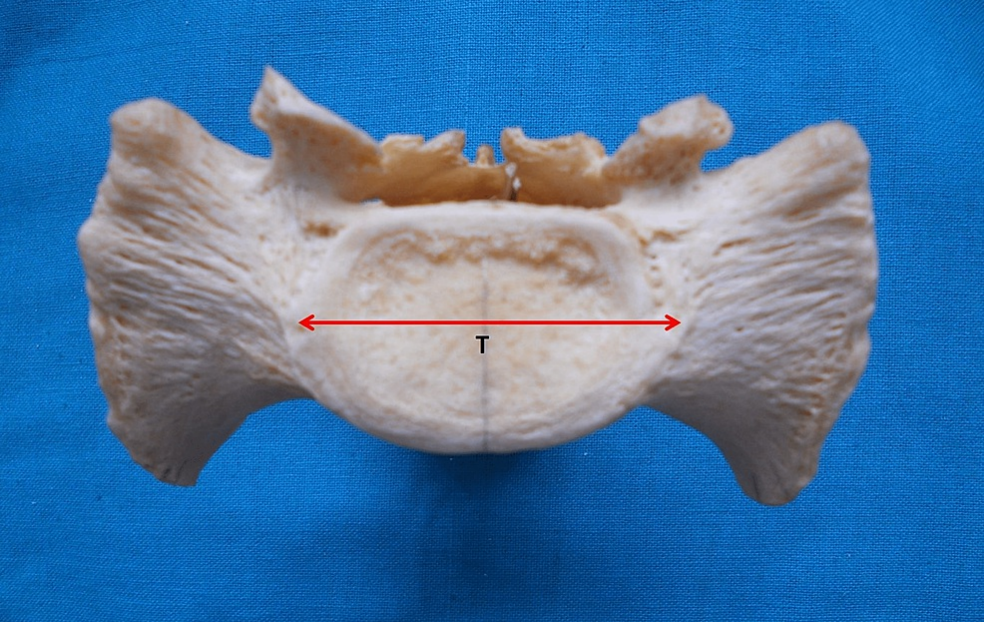
Superior aspect of the sacrum. T = transverse diameter of the S1 vertebral body

The transverse diameter of the S1 vertebral body was the greatest transverse dimension of the S1 vertebral body (Figure 2).

**Figure 3:**
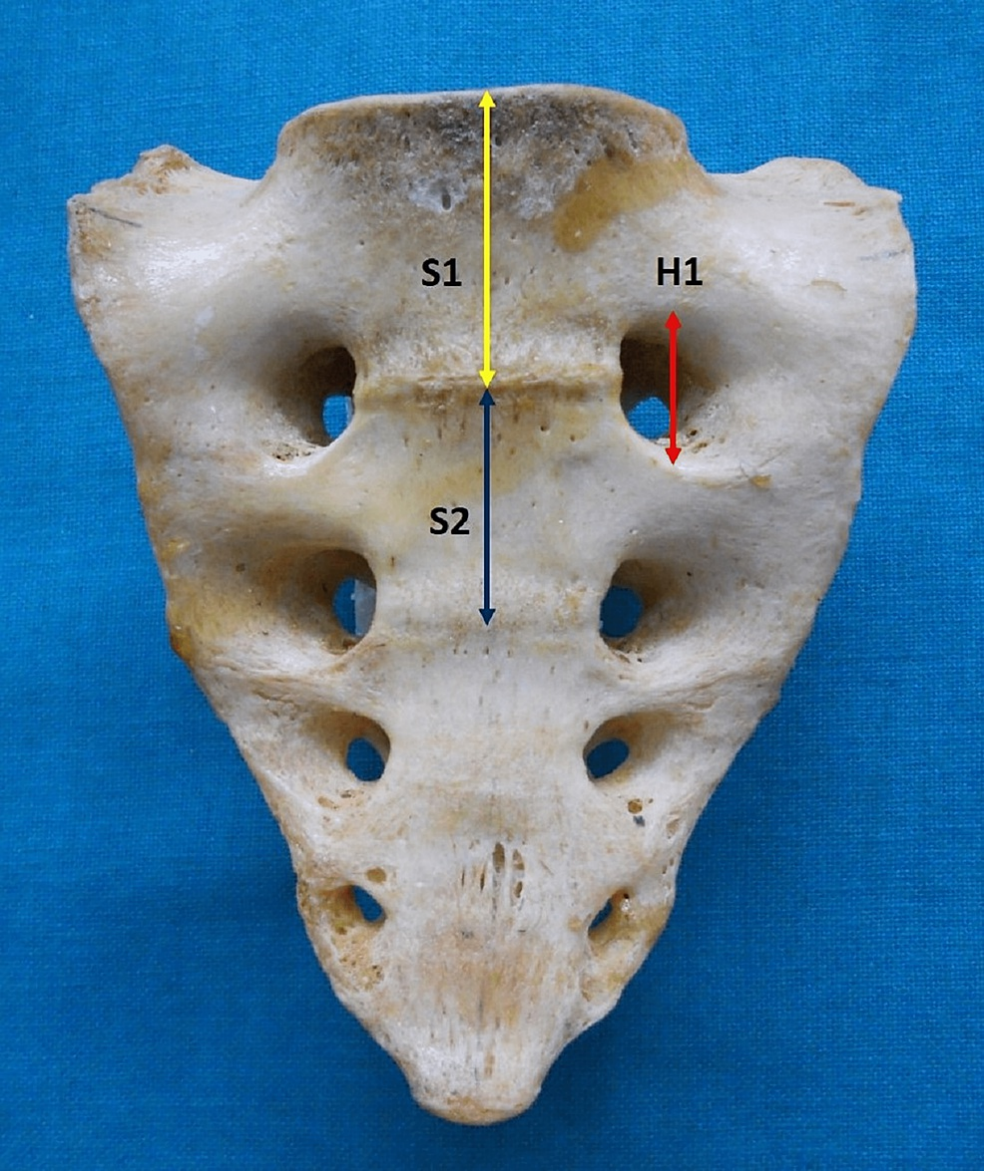
Anterior surface of the sacrum. S1 = height of the S1 vertebral body; S2 = height of the S2 vertebral body; H1 = height of the first anterior sacral foramina

The height of the S1 vertebral body was defined as the vertical distance between the mid-point of the transverse fusion line between the first anterior sacral foramina (ASF) and the mid-point of the promontory (Figure 3).

The height of the S2 vertebral body was the vertical distance between the mid-point of the transverse fusion line between the second ASF and the mid-point of the transverse fusion line between the first ASF (Figure 3).

**Figure 4:**
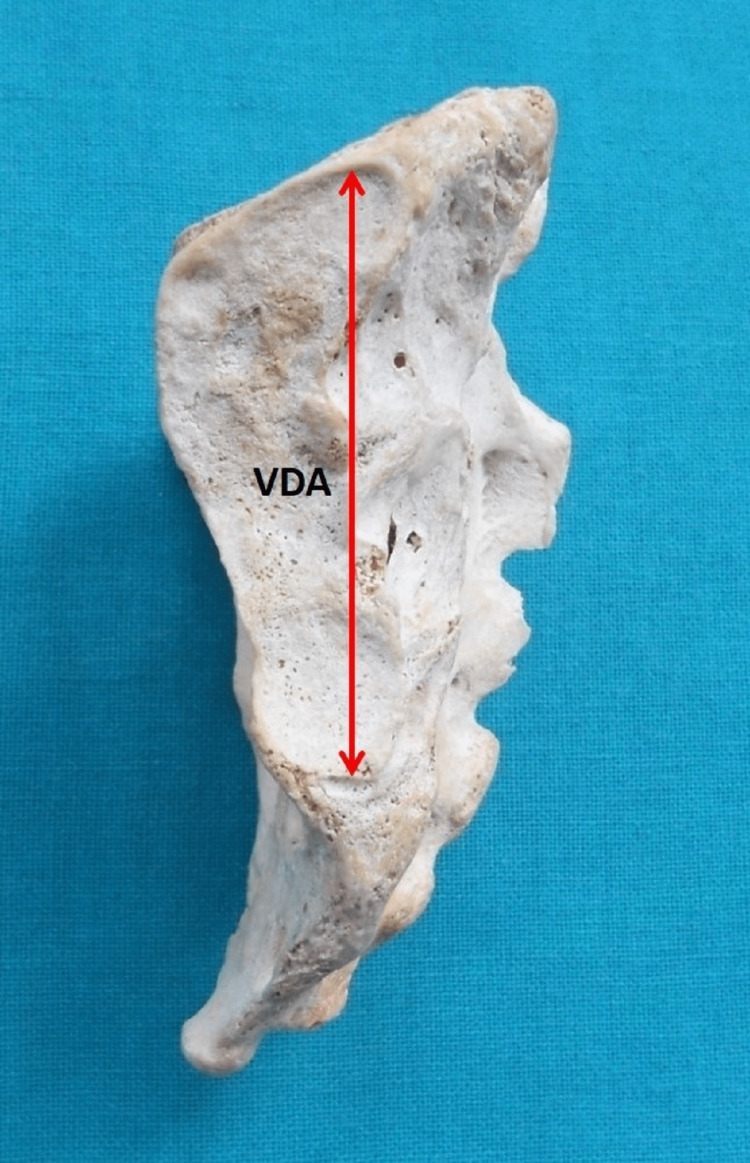
Lateral aspect of the sacrum. VDA = vertical diameter of the auricular surface

The vertical diameter of the auricular surface (VDA) was the highest vertical distance between two points, marked at the margin of the auricular surface (Figure 4).

The height of first ASF was the highest vertical distance between the upper and lower border of the first ASF (Figure 3).

**Figure 5:**
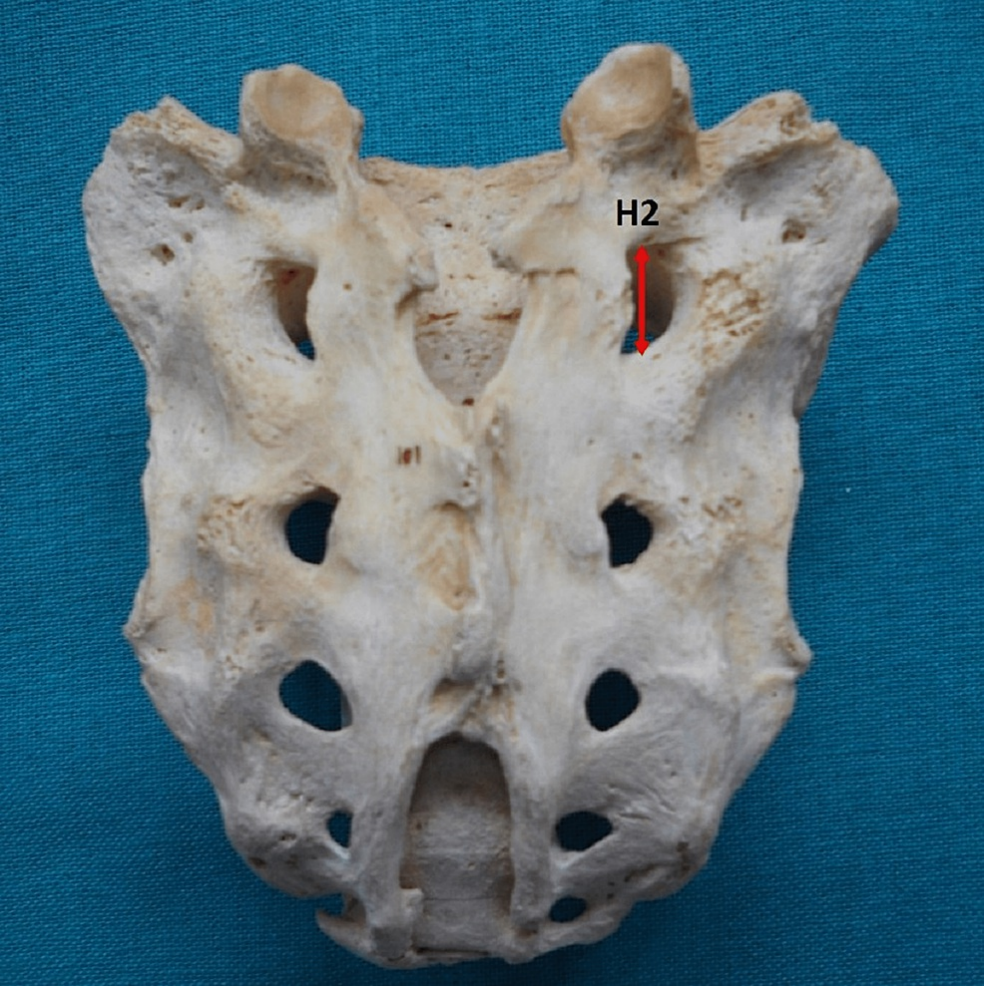
Posterior surface of the sacrum. H2 = height of the first posterior sacral foramina

The height of the first posterior sacral foramina (PSF) was the highest vertical distance between the upper and lower border of the first PSF (Figure 5).

Statistical analysis

SPSS version 17.0 (SPSS Inc., Chicago, IL, USA) was used for statistical analysis. Results were described as mean ± standard deviation (SD). Using Student’s t-test, morphometric dimensions of male and female sacra were compared and p-values were determined. A receiver operating characteristic (ROC) curve analysis was performed to determine the optimal cut-off values of various measurements. The area under the curve (AUC) with a 95% confidence interval (CI), sensitivity, and specificity was calculated.

ROC curve analysis

ROC curve analysis establishes a cut-off value that minimizes the number of false positives and false negatives which is the same as maximizing the sensitivity and specificity. Thus, a good choice for a morphometric parameter cut-off value is one which corresponds to a point on the ROC curve nearest to the upper left corner of the ROC graph. The ROC curves were calculated to illustrate true-positive results (sensitivity) versus false-positive results (1-specificity) as the cut-off for the morphometric parameter shifts from low to high. A morphometric parameter that yields no predictive information generates a straight line, whereas a parameter that discriminates well between the two groups will have a high rate of true-positive results and low rates of false-positive results yielding an elliptical curve. The accuracy is given by the area under the ROC curve.

## Results

A total of 110 dry adult human sacra were considered to study the sexual dimorphism in various morphometric parameters. The mean maximum breadth of the sacrum was greater in females, and this sex difference was statistically significant (p = 0.008). The mean sacral length measured from the promontory to the apex of the sacrum was higher in males, and this sex difference was highly significant (p < 0.001). Furthermore, the sacral index had higher values in female sacra than in male sacra (p < 0.001) (Table 1).

**Table 1:** Morphometric parameters in male and female sacra (in mm).

Parameter	Female	Male	P-value
Maximum breadth	106.95 ± 6.28	103.22 ± 7.40	0.008
Length	93.88 ± 8.09	107.60 ± 9.98	<0.001
Sacral index	114.35 ± 7.13	96.11 ± 6.42	<0.001
Transverse diameter of the S1 vertebral body	45.89 ± 5.23	47.45 ± 4.77	0.087
Height of the S1 vertebral body	29.04 ± 2.59	29.51 ± 2.54	0.265
Height of the S2 vertebral body	23.70 ± 2.77	25.64 ± 2.52	0.001

The mean TD and height of the S1 vertebral body were greater in the male sacra. No significant sex difference was observed in relation to the dimensions of the S1 vertebral body. The height of the S2 vertebral body was higher in the male sacra, which was highly significant (p = 0.001) (Table 1).

The mean VDA was higher in male sacra bilaterally, but it was statistically significant on the left side only (p = 0.032). The mean height of the first ASF was greater on both sides in the male sacra compared to the female sacra, and this difference was statistically highly significant (p = 0.005, right side; p = 0.003, left side). Similarly, the mean height of the first PSF was higher in male sacra bilaterally, and this was highly significant on the right side (p = 0.003, right side; p < 0.001, left side) (Table 2).

**Table 2:** Morphometric parameters in male and female sacra (in mm). VDA = vertical diameter of the auricular surface; ASF = anterior sacral foramina; PSF = posterior sacral foramina

Parameter	Right		P-value	Left		P-value
	Female	Male		Female	Male	
VDA	52.35 ± 4.44	53.89 ± 4.80	0.096	52.41 ± 4.82	54.55 ± 5.03	0.032
Height of the first ASF	12.38 ± 1.55	13.30 ± 1.63	0.005	12.42 ± 1.53	13.34 ± 1.54	0.003
Height of the first PSF	9.41 ± 2.67	11.02 ± 2.63	0.003	9.11 ± 2.60	11.05 ± 2.45	<0.001

ROC analysis of the maximum breadth of sacra

**Figure 6:**
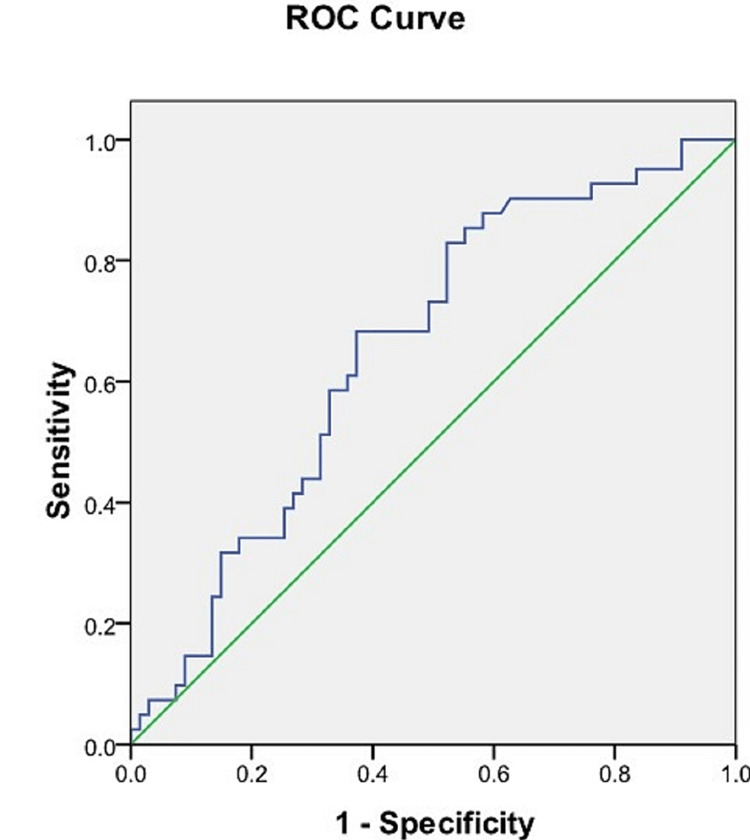
ROC curve of the maximum breadth of the sacrum. Blue line: ROC curve of the maximum breadth of the sacrum. Green line: Reference line. ROC = receiver operating characteristic

Using the ROC curve, a cut-off value of the maximum breadth of the sacra was calculated to be able to predict the sex of the sacra. A cut-off value of 101.38 mm was determined which was able to predict the sex of sacra with a sensitivity of 82.9% and a specificity of 47.8%. The area under the ROC curve was 0.654 (95% CI = 0.550-0.758) (Figure 6, Table 3).

**Table 3:** ROC curve analysis of morphometric parameters of the sacrum. ROC = receiver operating characteristic; TD = transverse diameter; VDA = vertical diameter of the auricular surface; ASF = anterior sacral foramina; PSF = posterior sacral foramina

Parameter	AUC	Sensitivity	Specificity	Accuracy	P-value
Maximum breadth	0.654	0.829	0.478	0.611	0.001
Length	0.862	0.78	0.85	0.824	< 0.001
Sacral index	0.994	0.976	0.97	0.972	< 0.001
TD of the S1 vertebral body	0.574	0.195	0.97	0.675	0.006
Height of the S1 vertebral body	0.563	0.39	0.686	0.592	0.053
Height of the S2 vertebral body	0.676	0.634	0.716	0.685	< 0.001
VDA	0.593	0.219	0.97	0.685	0.002
Height of the first ASF	0.669	0.537	0.791	0.694	< 0.001
Height of the first PSF	0.701	0.463	0.851	0.703	< 0.001

ROC analysis of the length of the sacrum

**Figure 7:**
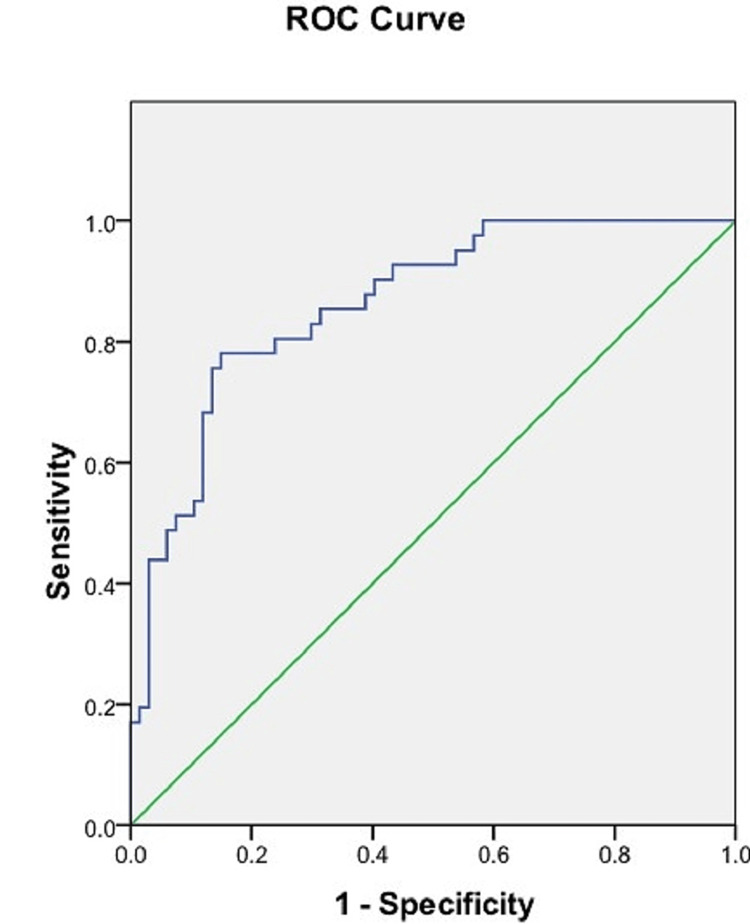
ROC curve of the length of the sacrum. Blue line: ROC curve of the length of the sacrum. Green line: Reference line. ROC = receiver operating characteristic

Using the ROC curve, the cut-off value of the length of the sacrum was calculated to differentiate between male and female sacra. A sacrum length ≤99.04 mm was able to predict the sex of the sacra with a sensitivity of 78% and a specificity of 85%. The AUC was 0.862 (95% CI = 0.794-0.931) (Figure 7, Table 3).

ROC analysis of the sacral index

**Figure 8:**
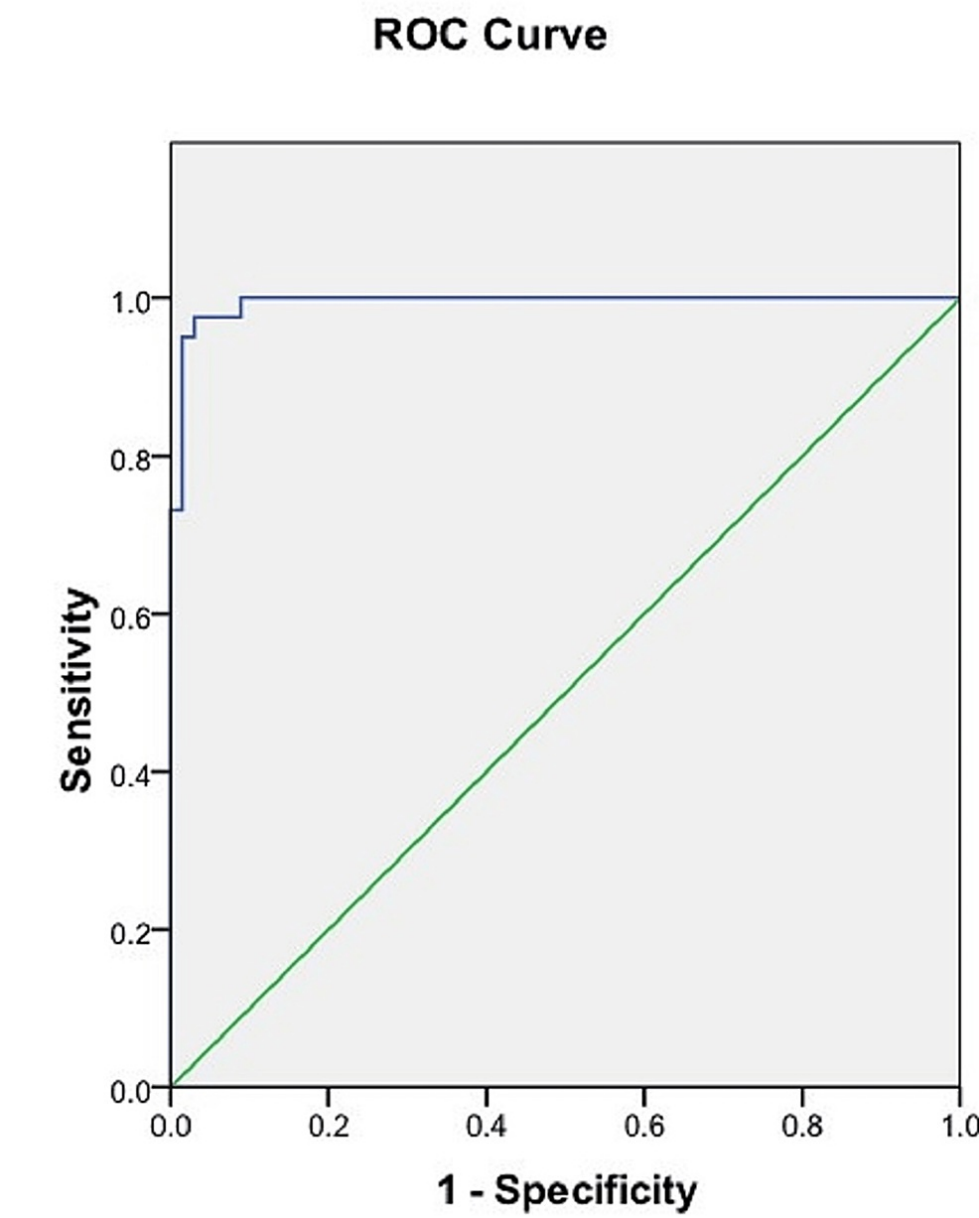
ROC curve of the sacral index. Blue line: ROC curve of the sacral index. Green line: Reference line. ROC = receiver operating characteristic

ROC analysis of the sacral index found a cut-off value of 105.85 mm which could discriminate well between male and female sacra. The accuracy per the area under the ROC curve was 0.994 (95% CI = 0.0-1.000). The cut-off value for the sacral index obtained had high sensitivity (97.6%) and specificity (97%) (Figure 8, Table 3).

ROC analysis of the TD of the S1 vertebral body

**Figure 9:**
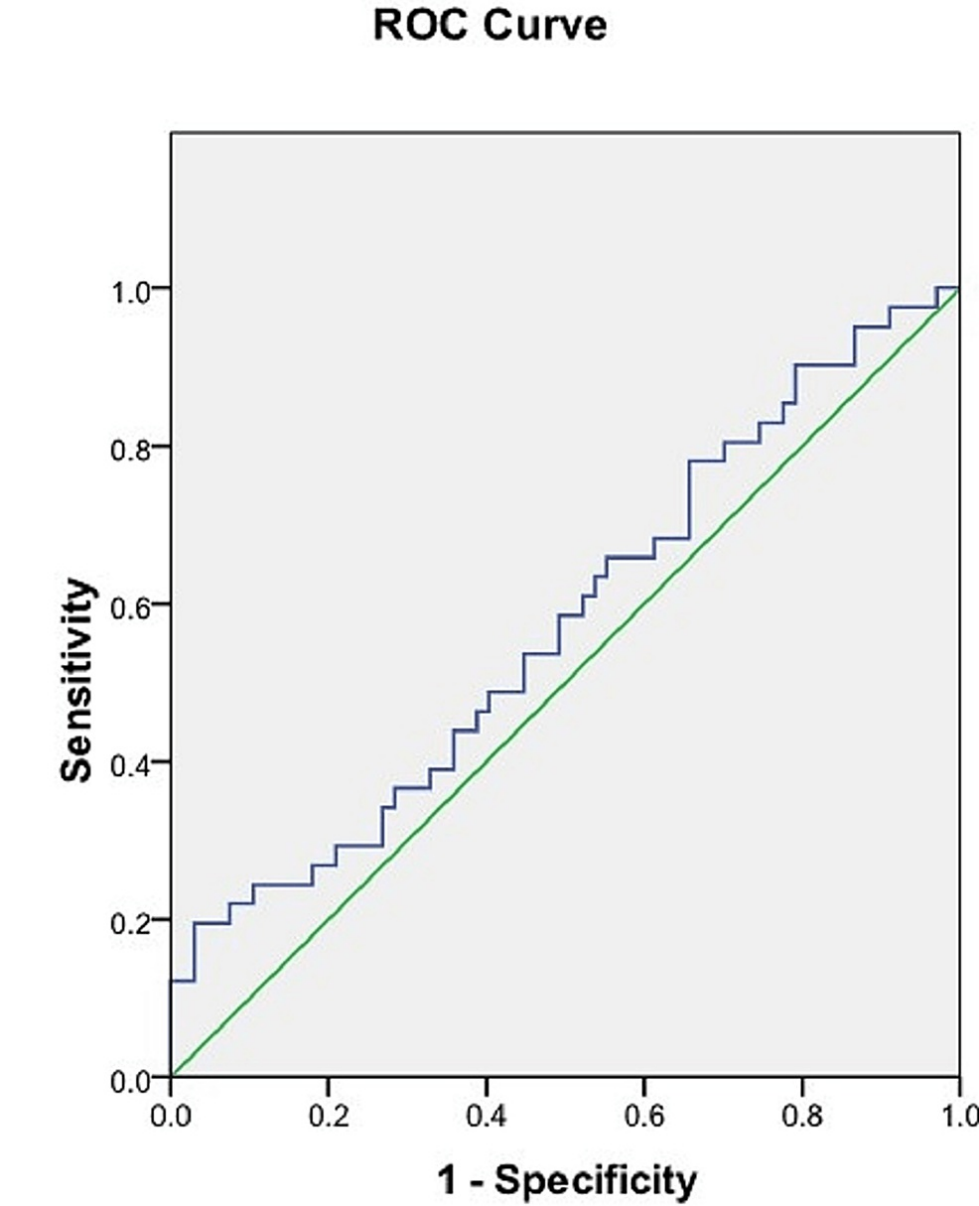
ROC curve of the transverse diameter of the S1 vertebral body. Blue line: ROC curve of the transverse diameter of the S1 vertebral body. Green line: Reference line. ROC = receiver operating characteristic

Using ROC analysis, a cut-off value of 40.435 mm for the TD of the S1 vertebral body was obtained which could differentiate between male and female sacra, with a sensitivity of 19.5% and specificity of 97%. The area under the ROC curve was 0.574 (95% CI = 0.462-0.686) (Figure 9, Table 3).

ROC analysis of the height of the S1 vertebral body

**Figure 10:**
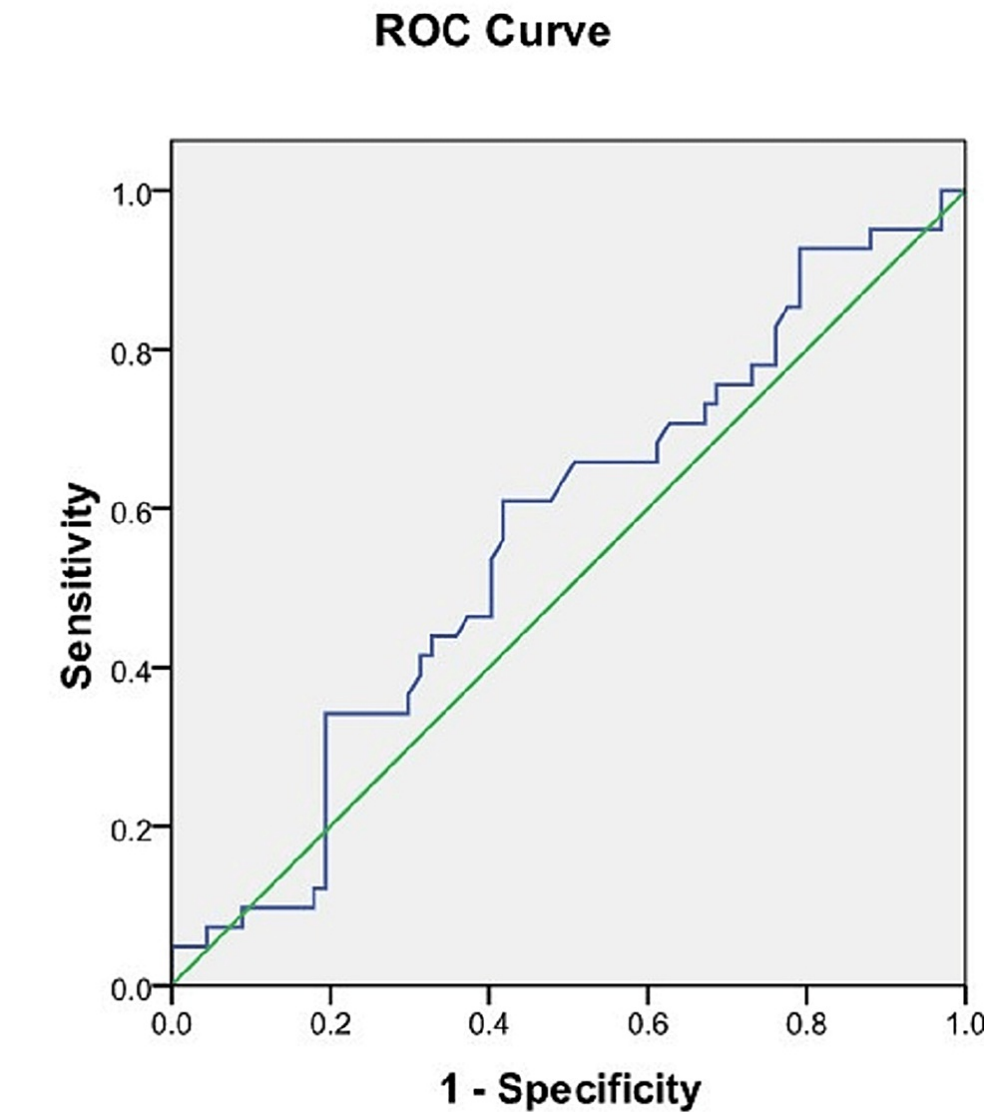
ROC curve of the height of the S1 vertebral body. Blue line: ROC curve of the height of the S1 vertebral body. Green line: Reference line. ROC = receiver operating characteristic

A cut-off value of 29.165 mm was calculated using the ROC curve for the height of the S1 vertebral body, which could predict the sex of the sacra. This value was able to differentiate between male and female sacra with a sensitivity of 39% and a specificity of 68.6%. The area under the ROC curve was 0.563 (95% CI = 0.452-0.674) (Figure 10, Table 3).

ROC analysis of the height of the S2 vertebral body

**Figure 11:**
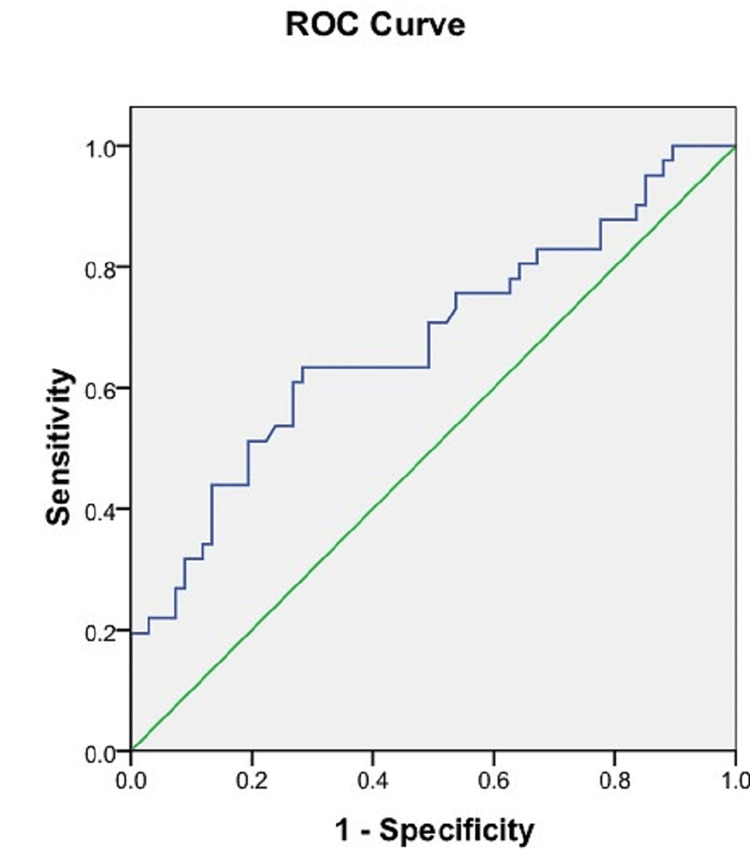
ROC curve of the height of the S2 vertebral body. Blue line: ROC curve of the height of the S2 vertebral body. Green line: Reference line. ROC = receiver operating characteristic

A cut-off value of 24.17 mm for the height of the S2 vertebral body was obtained using the ROC curve. The area under the ROC curve was 0.676 (95% CI = 0.568-0.784). This value had a high sensitivity and specificity of 63.4% and 71.6%, respectively (Figure 11, Table 3).

ROC analysis of VDA

**Figure 12:**
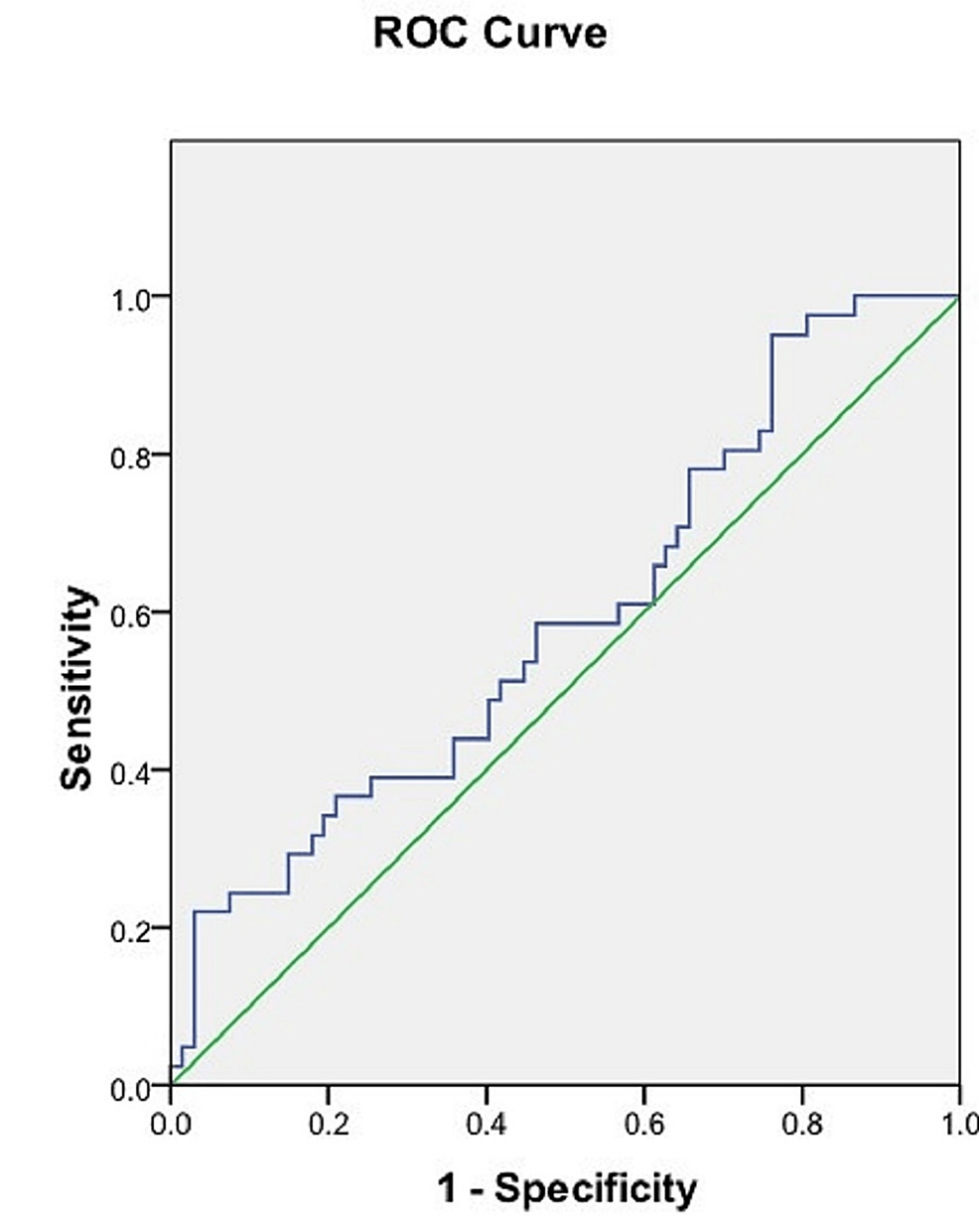
ROC curve of VDA. Blue line: ROC curve of VDA. Green line: Reference line. ROC = receiver operating characteristic; VDA = vertical diameter of the auricular surface

ROC analysis of VDA found a cut-off value of 47.503 mm which could differentiate between male and female sacra, and this difference was statistically highly significant (p = 0.002). The area under the ROC curve was 0.593 (95% CI = 0.482-0.704). The cut-off value of 47.503 mm was highly specific (97%) with low sensitivity (21.9%) (Figure 12, Table 3).

ROC analysis of the height of the first ASF

**Figure 13:**
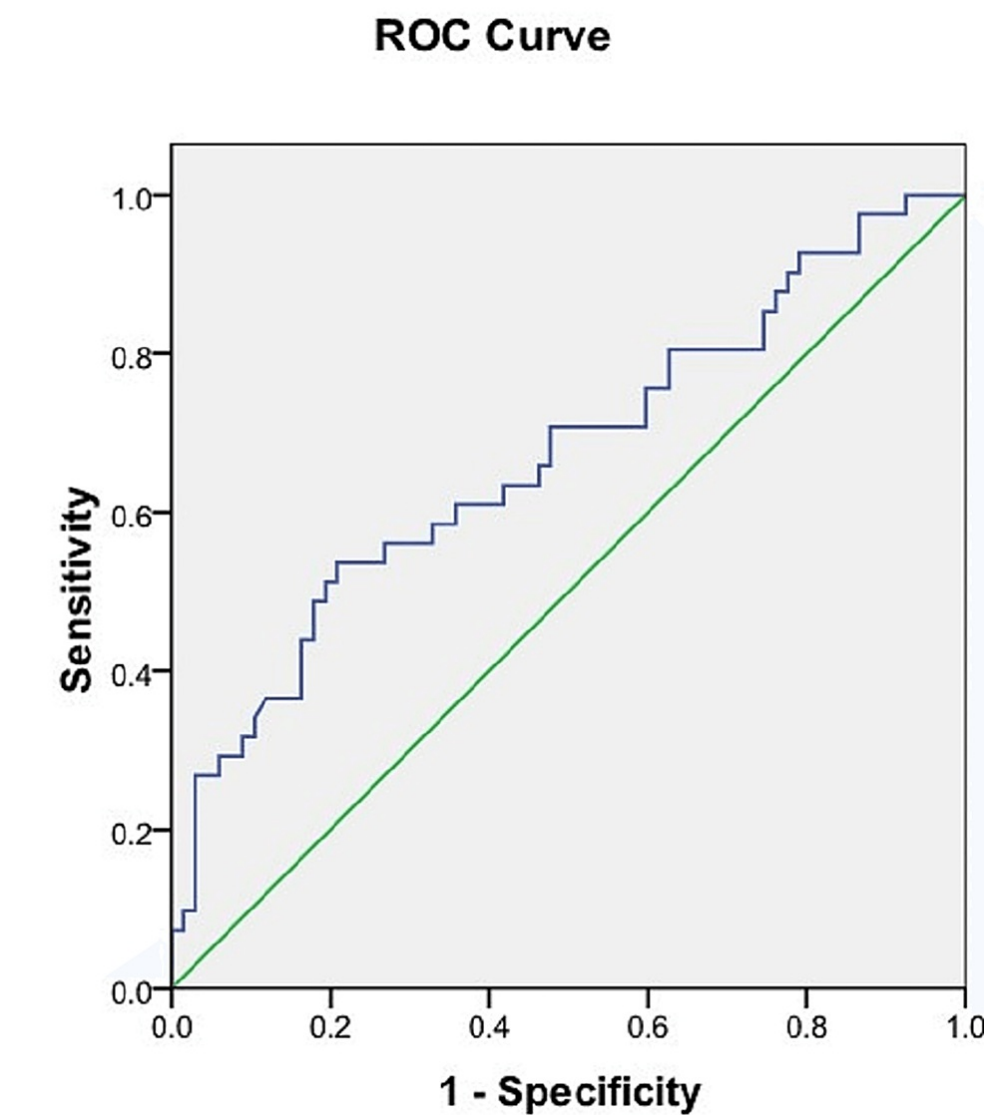
ROC curve of the height of the first ASF. Blue line: ROC curve of the height of the first ASF. Green line: Reference line. ROC = receiver operating characteristic; ASF = anterior sacral foramina

ROC curve found a cut-off value of 12.35 mm for the height of the first ASF which could predict sex difference in sacra, and this difference was statistically highly significant. This cut-off value of 12.35 mm had a sensitivity of 53.7% and a specificity of 79.1%. The area under the ROC curve was 0.669 (95% CI = 0.561-0.777) (Figure 13, Table 3).

ROC analysis of the height of the first PSF

**Figure 14:**
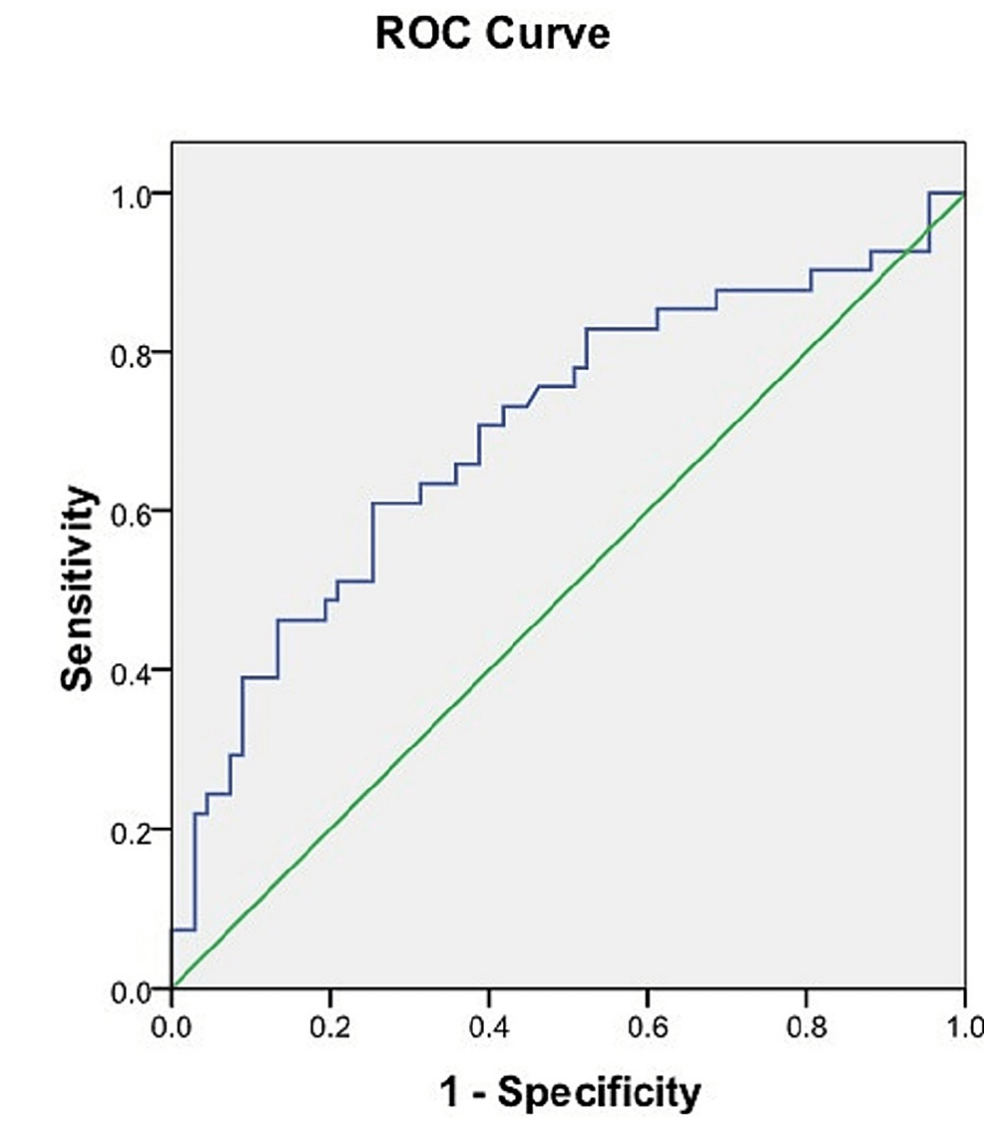
ROC curve of the height of the first PSF. Blue line: ROC curve of the height of the first PSF. Green line: Reference line. ROC = receiver operating characteristic; PSF = posterior sacral foramina

We found a cut-off value of 8.25 mm for the height of the first PSF, which could predict the sex of the sacra, and this was statistically highly significant. The accuracy as given by the area under the ROC curve was 0.701 (95% CI = 0.595-0.806). The cut-off value of 8.25 mm had a sensitivity of 46.3% and a specificity of 85.1% (Figure 14, Table 3).

## Discussion

Sex estimation is a crucial practice for forensic anthropologists. It is difficult to determine the sex of damaged or fragmented skeletal remains, especially after mass calamities [8,9]. Morphological methods for sex estimation are less reliable when compared to metric methods. Osteometric standards regarding sex estimation cannot be implemented in different populations as they vary with genetic and environmental factors and are linked with profession and lifestyle. Hence, it is essential to make temporary representative skeleton collections for population-specific anthropological standards [9,10]. Anatomists, forensic experts, and anthropologists constantly endeavor to discover new methods to determine more accurate identification of sex depending on different parts of the skeleton. The sacrum is a key bone as it is both the continuation of the vertebra and the pelvic girdle bones [9]. Hence, this study was conducted on morphometric parameters of the sacrum to investigate sexual dimorphism.

In this study conducted in an Indian population, the mean maximum breadth of the sacrum was greater in females which was comparable to the findings of Isaac et al. in a Nigerian population, Kumar et al. in an Arabian population, Bakici et al. in a Turkish population, and Mishra et al., Arora et al., Kumar et al., and Parashuram et al. in an Indian population [2,5,9,11-14] (Table 4). Other researchers including Elkhateeb et al. in an Egyptian population and Sachdeva et al., Ravichandran et al., and Bhanarkar et al. in an Indian population reported lower values in female sacra [1,4,15,16] (Table 4). In the ROC analysis, we observed that the maximum breadth had a lesser AUC value of 0.654, which is comparable to the finding of Bakici et al. (AUC value = 0.55). Hence, it can be stated that the maximum breadth alone is not a good parameter for differentiating the sex of sacra.

**Table 4:** Comparison of various parameters of the sacrum with other studies.

Study	Population	Sex	Breadth (mm)	Length (mm)	Sacral index	Transverse diameter of the S1 vertebral body (mm)
Sachdeva et al. [1]	Indian	Male	103.1	104.1	100.24	47.6
		Female	101.7	91.8	111.74	45.5
		P-value	0.612	0.005	0.016	0.380
Arora et al. [2]	Indian	Male	101.94	109.74	93.68	-
		Female	114.13	91.22	125.35	-
		P-value	<0.0002	<0.0001	<0.0001	-
Elkhateeb et al. [4]	Egyptian	Male	114	109	-	57
		Female	112.9	100.3	-	55.9
		P-value	<0.197	<0.05	-	0.155
Kumar et al. [5]	Arabian	Male	106.8	102.7	97.51	53
		Female	117.1	93.5	117.35	51.4
		P-value	<0.0001	<0.0001	<0.001	0.0632
Bakici et al. [9]	Turkish	Male	113.6	110.3	-	28.9
		Female	114.6	105.5	-	32.3
		P-value	0.46	0.01	-	<0.05
Isaac et al. [11]	Nigerian	Male	115.9	109.2	106.3	-
		Female	116.3	103.5	112.2	-
		P-value	>0.05	>0.05	<0.05	-
Mishra et al. [12]	Indian	Male	105.34	107.53	98.21	49.12
		Female	105.79	90.58	117.84	42.81
		P-value	>0.05	<0.001	<0.001	<0.001
Kumar et al. [13]	Indian	Male	101.53	104.55	97.66	46.53
		Female	105.67	94.66	112.12	30.85
		P-value	<0.001	<0.0001	<0.0001	>0.05
Parashuram et al. [14]	Indian	Male	103.8	102.68	101.26	-
		Female	105.57	91.11	116.18	-
		P-value	>0.05	<0.001	<0.001	-
Ravichandran et al. [15]	Indian	Male	93.7	97.8	96.32	-
		Female	92.91	90.96	102.29	-
		P-value	<0.0001	<0.0001	<0.0001	-
Bhanarkar et al. [16]	Indian	Male	97.4	104.8	99.3	42.8
		Female	96.2	90.8	111.2	40.3
		P-value	<0.009	<0.011	<0.001	<0.021
Present study	Indian	Male	103.22	107.60	96.11	47.45
		Female	106.95	93.88	114.35	45.89
		P-value	0.008	<0.001	<0.001	0.087

The mean sacral length was greater in males in this study which was analogous to the observations of other investigators (Table 4). In the ROC analysis, the AUC value for the length of the sacra from the promontory to the apex was 0.862 in our study while it was 0.63 in the study conducted by Bakici et al. in an Egyptian population (Table 4). Therefore, the length of the sacra can be considered a good parameter for sex estimation, particularly in the Indian population.

The sacral index was greater in females in our study similar to the findings of other researchers (Table 4). In the ROC analysis, the highest values for AUC (0.994) and accuracy (0.972) were observed for the sacral index among all parameters considered for sex determination. Hence, the sacral index can be an excellent parameter for the identification of the sex of the sacra.

TD of the S1 vertebral body was more in males in this study which is comparable to the findings of other investigators (Table 4). In the ROC analysis, the AUC value for TD of the S1 vertebral body was 0.574. Therefore, the TD of the S1 vertebral body cannot be considered a good parameter for the estimation of the sex of the sacra.

We could not find any research on ROC analysis of sacral parameters such as the height of the S1 and S2 vertebral body, VDA, and the height of the first ASF and the first PSF after thoroughly reviewing the literature. However, in this study, ROC analysis of these parameters revealed fair values for the height of the S2 vertebral body (AUC = 0.676; p <0.001), the height of the first ASF (AUC = 0.669; p <0.001), and the height of the first PSF (AUC = 0.701; p <0.001) for the identification of the sex of the sacra in Indian population (Table 3). Hence, these parameters can be utilized to identify the sex with an accuracy of 60-70% even if only fragmented sacra are available.

This study was performed on 110 dry adult human sacra due to the shortage of bones in the institute. We propose further investigations on other morphometric parameters on a large number of sacra which could be crucial, especially when only fragmented bone is available for sex estimation.

## Conclusions

This study emphasizes the significance of various morphometric parameters in the determination of the sex of the sacra. In the ROC analysis, the sacral index was observed to be the best morphometric parameter for the identification of the sex of the sacra with the highest AUC value (0.994) and the highest accuracy (0.972). The length of the sacra is another good parameter for establishing sex (AUC = 0.862; accuracy = 0.824). Furthermore, the height of the S2 body, the height of the first ASF, and the height of the first PSF can be considered with an accuracy of 60-70% if only a fragmented sacrum is obtainable for sex estimation. This study highlights the importance of morphometric parameters of the sacrum in sex estimation, especially in medicolegal cases when the skull and pelvis are shattered or unobtainable. We anticipate that this study will be able to offer a reference database of morphometric parameters for the identification of the sex of sacra in the Indian population.
